# Malaria Prevalence and Risk Factors in Outpatients at Teda Health Center, Northwest Ethiopia: A Cross-Sectional Study

**DOI:** 10.1155/2024/8919098

**Published:** 2024-05-14

**Authors:** Dagmawi Woldesenbet, Yalewayker Tegegne, Muluken Semaw, Wagaw Abebe, Silesh Barasa, Menberu Wubetie, Elias Tamene, Marshet Anteneh, Aschalew Yimer, Deneke Wolde

**Affiliations:** ^1^Department of Medical Laboratory Science, College of Medicine and Health Science, Wachemo University, Hossana, Ethiopia; ^2^Department of Medical Parasitology, School of Biomedical and Laboratory Science, College of Medicine and Health Sciences, University of Gondar, Gondar, Ethiopia; ^3^Medical Laboratory Unit, Sanja General Hospital, Amhara National Regional State Health Bureau, Sanja, Ethiopia; ^4^Department of Medical Laboratory Science, College of Health Science, Woldia University, Woldia, Ethiopia; ^5^Department of Medical Laboratory Science, College of Medicine and Health Science, Arba Minch University, Arba Minch, Ethiopia; ^6^Department of Medical Laboratory Science, College of Medicine and Health Science, Dire Dawa University, Dire Dawa, Ethiopia; ^7^Bahir Dar Blood Bank, Amhara National Regional State Health Bureau, Bahir Dar, Ethiopia; ^8^Medical Laboratory Unit, Teda Health Center, Amhara National Regional State Health Bureau, Gondar, Ethiopia

## Abstract

**Background:**

Despite extensive efforts and achievements made in prevention and control, malaria is still a public health problem in Ethiopia. Currently, the case number has been climbing, even changing the epidemiology to the previously low-burden reporting locations. Therefore, our study is aimed at assessing malaria prevalence and associated risk factors in outpatients, Northwest Ethiopia.

**Methods:**

A facility-based cross-sectional study was conducted among 318 malaria-suspected outpatients from October 22 to December 15, 2022, at Teda Health Center, Northwest Ethiopia. Sociodemographic characteristics and malaria risk factors were assessed by a face-to-face interview using a pretested questionnaire. *Plasmodium* parasites were detected by using microscopy on a blood film stained with 10% and 3% Giemsa stain. The data were analyzed using Statistical Package for Social Sciences software version 25, and statistical significance was reported at a *p* value < 0.05.

**Results:**

Of the 318 study participants, 124 (39.0%; 95%CI = 33.6% − 44.6%) tested positive for *Plasmodium* infection. *Plasmodium vivax*, *P. falciparum*, and mixed infections were identified in 71 (57.3%), 47 (37.9%), and 6 (4.8%), respectively, among positive study participants. Individuals above the age of 15 (AOR = 2.704, 95% CI 1.370-5.336) were more likely to be infected with *Plasmodium* than under-five-year-old individuals. Rural residents (AOR = 2.56, 95% CI 1.281-5.098), those who sleep or work outside at night (AOR = 1.99, 95% CI 1.020-3.875), and individuals who did not use long-lasting insecticide-treated net (AOR = 3.26, 95% CI 1.633-6.499) and had a family member with a history of malaria (AOR = 2.902, 95%CI = 1.671 − 5.041) had higher odds of acquiring *Plasmodium* infection than their counterparts.

**Conclusion:**

In our study area, malaria was a major health problem, with *P. vivax* being the dominant species. Multiple environmental and behavioural factors were associated with the infection acquisition. Therefore, urgent holistic intervention is required.

## 1. Background

Malaria is a haematozoan parasitic disease caused by *Plasmodium* species and transmitted by the bite of infected female *Anopheles* mosquitoes, usually during twilight and sunrise. Malaria-causing *Plasmodium* species differ in morphology, host interactions, and clinical manifestations. These are *Plasmodium (P) falciparum*, *P. vivax*, *P. ovale*, *P. malariae*, and *P. knowlesi*. The first two are the dominant species and leading causes of severe malaria [1].

The disease is serious and spread in 87 countries, comprising more than 3 billion people [1]. According to the World Health Organization (WHO) malaria report, approximately 249 million cases and 608,000 deaths were reported worldwide in 2022. The worldwide malaria burden is disproportionately high in Africa, with 93.6% and 95.4% of global cases and deaths, respectively. In 2022, Ethiopia was one of the major contributors to the global rise of malaria from the previous year, with a 35% increase in the cases number. The report indicated an estimated 2.4 million malaria cases and 180 reported deaths [2].

Malaria affects over 75% of Ethiopia's landmass, and approximately 68% of the Ethiopian population lives in malaria-risk areas [3, 4]. It remains a leading public health problem in the country due to low coverage of long-lasting insecticide-treated bed nets (LLIN), low coverage of indoor residual spraying (IRS), drug resistance, insecticide resistance of malaria vectors, poor access to health care, migration of people from malaria-endemic areas to nonendemic areas, and false microscopic results [5].

In Ethiopia, malaria transmission occurs predominantly at elevations below 2000 m, with altitude and rainfall appearing to be extremely significant factors. However, locations with altitudes above 2000 m have reported an increase in malaria burden [6, 7]. The previous reports that showed malaria being found at low levels in the highlands made the attention given to these areas lower [8, 9]. The disease transmission pattern is diverse and unstable, with epidemics occurring in a variety of locations throughout the country [10]. The main transmission season in most parts of the country is from September to December, coinciding with crop harvesting time, following the main rainy season from June to September, while a minor transmission season occurs between April and May [11, 12].

The number of malaria cases had declined consistently since 2000 in Ethiopia [13–15]. Following this, Ethiopia planned to eliminate malaria by 2030 by implementing a variety of interventional measures, including early detection and treatment, selective vector control measures such as IRS and LLIN, and environmental management. Additionally, rapid diagnostic tests are performed, along with the adaptation of artemisinin-based drugs [5]. However, different transmission dynamics and trend analysis studies have elucidated that the number of cases is rising in Ethiopia [16–19]. The emergence and reintroduction of malaria in previously eliminated or reduced locations is becoming a new challenge for malaria elimination efforts [20].

In recent years, malaria incidence has increased in specific settings. Integrating strategy is warranted for the reduction of the malaria burden and achieving the goal of malaria elimination [15]. As a result, consistent assessments of malaria prevalence in endemic areas aid in evaluating existing intervention strategies and customising local intervention methods. Besides, the risk factors for malaria in the highlands need to be studied for the targeted adjustment of interventions. Moreover, there is also a paucity of studies showing the malaria burden in this study area. Understanding the malaria prevalence and its possible risk factors is mandatory for all-level control management. The prevalence should be studied at a small focal level for the local-level adjustment of prevention strategies and interventions. Therefore, we aimed to determine the prevalence of malaria and associated risk factors in outpatients at Teda Health Center, Northwest Ethiopia.

## 2. Materials and Methods

### 2.1. Study Design and Period

A facility-based cross-sectional study design was conducted from October 22 to December 15, 2022.

### 2.2. Study Area

The study was conducted at Teda Health Center, Northwest Ethiopia. The health center is found in Teda subcity, which is located 29 km away in the Gondar City administration, Amhara National Regional State. The health center serves more than 20,000 people from Teda and *Kebeles* nearby. Teda is located 698 km from Addis Ababa, the capital of Ethiopia. It is located at 11° 20′ 57.93^″^ N latitude and 37° 58′ 42.45^″^ E longitude, 2,200 meters above sea level. The annual temperature of the area ranges from 22°C to 29°C, with the warmest and coldest months being March and July, respectively. And the area receives about 1,162 mm of annual rainfall. There are many streams and a dam, which is currently under construction and is considered the major breeding site for mosquitoes by the local administrators. The livelihood of the population is almost entirely dependent on farming. The main crops produced in the subcity include *teff* (*Eragrostis tef*), red highland sorghum (*Sorghum bicolor*), maize (*Zea mays*), and barley (*Hordeum vulgare*). The Teda population is perennially infected with malaria, and the number of cases rises twice a year in the major transmission season from September to December and the minor transmission season from April to June. Especially during these seasons, the health center is known for having frequent malaria patients. It is the leading health facility in Gondar town for diagnosing and treating multiple cases of malaria (*Unpublished Gondar town health office report*).

### 2.3. Sample Size Calculation and Sampling Technique

The sample size was calculated using the single population proportion formula by taking the 25.1% malaria prevalence at Maksegnit Health Center in Northwest Ethiopia [16], a 95% confidence interval, a 5% margin of error, and a 10% nonresponse rate.

(1) $$\begin{array}{c} n=\frac{p\left(1-p\right){\left(z\alpha /2\right)}^{2}}{d^{2}}=\frac{0.251\left(1-0.251\right){\left(1.96\right)}^{2}}{0.05^{2}}=289+289\left(10\%\right)\approx 318. \end{array}$$

Patients who met the eligibility criteria were selected by systematic random sampling, considering the case flow of the health center for malaria diagnosis from September to December of 2021, which were 4,017 individuals. The case number (4,017) was divided by the sample size (318), and every 13^th^ patient was included in the study.

### 2.4. Study Population

The study populations were all malaria-suspected outpatients with cardinal signs and symptoms of malaria (having fever, chills, and headache) who attended the health center during the study period.

### 2.5. Eligibility Criteria

Individuals who were residents of the study area or lived within the previous six months were included. Those who were severely ill and/or unable to respond to the study questions and who take antimalarial drugs with in the previous 30 days were excluded from the study.

### 2.6. Data Collection Methods

Following a briefing on the study's purpose, all participants provided informed consents and/or assents prior to the start of data and sample collection. A structured questionnaire that was developed from malaria prevalence related studies was used to collect sociodemographic, clinical, and infection-related factor data. The questionnaire was pretested using 5% of the sample size in the study area to assess clarity and identify any necessary amendments. The questionnaire was administered through a face-to-face interview before blood sample collection by well-trained health professionals using their mother tongue, Amharic. Attendants (mothers/fathers) were interviewed for children below the age of 15 years. An axillary temperature was measured twice using a calibrated thermometer, and the average was taken.

Twenty microliters of capillary blood was collected from a finger puncture, and then, paired thick and thin blood smears were made on microscopic slides. Thin blood films were fixed with absolute methanol. Then, both thin and thick films were stained with 10% and 3% Giemsa stain for 10 minutes and 30 minutes, respectively, following the procedure described elsewhere [21]. The thick films were examined under an oil immersion objective light microscope for the detection of *Plasmodium* parasites, and the thin films were examined for species identification when the thick films were found positive. An independent laboratory technologist confirmed the report of each 10% Giemsa-stained blood film result by 3% Giemsa-stained blood films.

### 2.7. Data Analysis

Data were double-entered in Excel for cleaning before being entered into Statistical Package for Social Science software version 25 for analysis. Mean, frequency, and percentage were used to describe the characteristics of the study participants. The association between risk factors and malaria infection was assessed by logistic regression. The crude odds ratio (COR) was determined by bivariate regression, and the adjusted odds ratio (AOR) was analyzed by multivariate logistic regression to measure the strength of the association between variables, and a *p* value of < 0.05 was considered statistically significant.

## 3. Results

### 3.1. Sociodemographic Characteristics

Of the total 318 study participants, 173 (54.4%) were males, and the mean age was 20.9 (standard deviation: 16.65) years. The majority of the study participants, 143 (45%), had primary education, followed by secondary education (84 or 26.4%), no formal education (73 or 23%), and college and above (18 or 5.6%). Among the study participants, 225 (70.8%), 82 (25.8%), 9 (2.8%), and 2 (0.6%) were single, married, widowed, and divorced, respectively. Most of the study participants were students, 125 (39.3%), followed by nonworkers, farmers, housewives, merchants, private employees, government employees, and labourers, 63 (19.8%), 33 (10.4%), 33 (10.4%), 25 (7.9%), 19 (6.0%), 10 (3.1%), and 10 (3.1%), respectively (Table 1).

### 3.2. Clinical Manifestations

Most of the clinical manifestations of malaria were seen in the under-five age group of study participants, with all of them having loss of appetite and weakness and 97.7% having an axillary temperature above 37.5°C. Of the total 318 study participants, headaches (90.9%) and loss of appetite (90.6%) were the most commonly manifested clinical manifestations (Table 2).

### 3.3. Malaria Prevalence and Risk Factors

**Figure 1:**
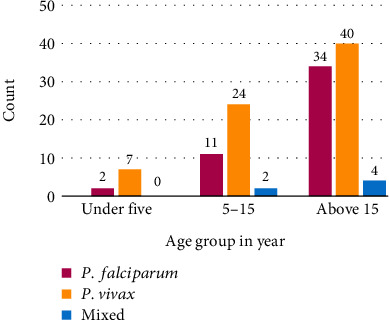
*Plasmodium* species distribution among different age groups at Teda Health Center, Northwest Ethiopia.

Malaria asexual parasite was detected in 124 (39.0%; 95%CI = 33.6% − 44.6%) study participants. *Plasmodium vivax* was found the dominant parasite species in this study area with 71 (57.3%) followed by *P. falciparum* with 47 (37.9%) and mixed infection of 6 (4.8%). *Plasmodium vivax* was dominant in all age groups (Figure 1).

The prevalence of malaria was 46.8% in males and 29.7% in females. The entire study's participants responded that their house had not been sprayed with insecticidal spray within the previous 12 months. Of the total 318 study participants, 106 (33.3%) had a history of malaria infection within the previous 12 months. The proportion of LLIN possessed by all study participants was 69.5%, and the majority of the study participants (67.6%) live near surface water within 500 m of their house. The surface water bodies were comprised of stagnant water, rivers, and a man-made dam (Table 3).

### 3.4. Factors Associated with Malaria Infection

In the multivariate logistic regression analysis, individuals in the above-15-year-old age group were 2.7 times more likely to be infected with malaria than those in the under-five year-old age group (AOR = 2.704, 95%CI = 1.370 − 5.336). The rural residents were 2.5 times more likely to be infected with the *Plasmodium* parasite than those who live in urban areas (AOR = 2.556, 95%CI = 1.281 − 5.098). Individuals who did not use LLIN were 3.2 times more vulnerable than individuals who used it for *Plasmodium* infection (AOR = 3.257, 95%CI = 1.633 − 6.499). Malaria risk was 2.9 times higher in individuals who had a family member with malaria than in those who did not (AOR = 2.902, 95%CI = 1.671 − 5.041). Individuals who sleep or work outside at night were about 1.9 times more likely to be infected with malaria than those who did not (AOR = 1.988, 95%CI = 1.020 − 3.875) (Table 4).

## 4. Discussion

Malaria continued to be a major public health problem widespread throughout tropical and subtropical regions of the world, especially in the WHO African region, including Ethiopia [3]. This study shows that malaria remains one of the most important public health concerns in Teda subcity, Gondar, Northwest Ethiopia. This study reported 39.0% malaria prevalence. The age of the study participants, residence, LLIN utilization, habit of sleeping or working outside at night, and presence of family members with a history of malaria were identified as the determinants of malaria infection.

The overall malaria burden of this study is consistent with the study conducted in Yeki District, Ethiopia (38.2%) [22] and Nigeria (41.6%) [23]. However, it is higher than other studies conducted in Northwest Ethiopia: Dembiya 3.5% [24], Lake Tana and its surrounding area 24.7% [25], Bahir Dar Zuria District 12.8% [26], Jawi District 16.4% [27], and Hamusit 29.0% [28]. It is also higher than the studies conducted in different settings in Ethiopia [29–31]. This might be due to the changes in the epidemiological transmission of malaria from the lowlands to the highlands of Ethiopia when temperatures rise in the highlands. In contrast, this study's malaria burden is lower than the studies conducted in Limmu District 49.4% [32], West Shoa Zone 47.7% [33], and Guba District 51.04% [34], Ethiopia. Previously, the Ethiopian Public Health Institute designated areas above 2000 m altitude as malaria-free zones, although not totally malaria-free at the district level [35]. However, this study result and other studies reported high malaria burden in areas with such altitude [15, 16, 26, 36].

These inconsistencies could be attributed to geographical differences and the seasonality of infection. Some areas may have scattered seasonal microgeographic local transmissions due to local environmental fitness. In addition, variation in the implementation and intensity of malaria intervention initiatives, as well as differences in the local epidemiology of malaria parasites, could be the factor. Moreover, it might be due to the difference in community knowledge about malaria transmission, prevention, and control.

In this study, *P. vivax* was the dominant species, followed by *P. falciparum* and a mixed infection of the two. This is inconsistent with the report of the WHO, which reported that *P. falciparum* is the dominant parasite in many parts of Ethiopia [3]. The dominance of *P. falciparum* was described in multiple studies conducted across Ethiopia [25, 27, 28]. However, reports showed that *P. vivax* was the dominant cause of malaria in various parts of Ethiopia [29, 37–41]. This is also in line with a study in India that reported 64% and 34% of *P. vivax* and *P. falciparum*, respectively [42]. This difference could be attributed to the study areas' different altitudes, where *P. falciparum* predominates in the lowlands while *P. vivax* predominates in the highlands of Ethiopia [43]. This might be linked to the capacity of *P. vivax* to survive in colder climates (higher altitudes) than other *Plasmodium* species [44]. In addition, the higher prevalence of *P. vivax* could be due to the rise of drug resistance against chloroquine and the relapsing nature of *P. vivax* [10]. The antirelapse therapy of *P. vivax* malaria should be supervised since it has a longer treatment regimen, which minimizes compliance with the drug, than the curative treatment of chloroquine.

In this study, individuals in the age group above 15 years were highly vulnerable to malaria. This is in agreement with studies conducted in Ethiopia [24, 41, 45]. This might be because these age group individuals are actively involved in outdoor activities such as agriculture and cattle herding in the evening, which makes them exposed to outdoor *Anopheles* mosquito bites [46]. The habit of sleeping or working outside at night as a risk factor for malaria infection in this study is in agreement with the study conducted in Dembiya District, Ethiopia [47]. These outdoor activities at night were predictors associated with malaria transmission in this study area. Outdoor sleeping or working activities were reported as determinants for the transmission of malaria in other studies conducted elsewhere [48, 49].

Rural residence and having a family member with history of malaria were also risk factors for malaria prevalence in this study area. This is in agreement with studies conducted in East Shewa, Ethiopia [41]; Hararge, Ethiopia [50]; and elsewhere [51–53]. The disease is more prevalent in rural areas due to favourable environmental conditions for the establishment and proliferation of vectors [41, 54]. Rural residence is also associated with poor housing quality, poor drainage systems, lower awareness about malaria transmission, and large family size, which were previously described as risk factors for malaria infection [24, 27, 29]. Indoor mosquito bites in the evenings while sitting inside their open houses or via holes might be the reason for the higher malaria burden among rural residents. Moreover, individuals who had a family member with malaria were more vulnerable to malaria than their counterparts. This is consistent with other studies conducted in Hamusit [28] and Shewa Robit [29], Ethiopia, and elsewhere [55]. This could be due to the interrupted biting behaviour of *Anopheles* mosquitoes more than one individual at the same time in the same house.

Utilization of LLIN was a significant protective factor against malaria infection in this study. This is in agreement with previous studies in Ethiopia [25, 56]. This is because LLIN either mechanically inhibits mosquitoes from biting or biologically kills mosquitoes that come into contact with it [57]. The use of LLIN is considered one of the paramount malaria prevention mechanisms. This demonstrates that there is a clear need to improve the strategies of LLIN distribution so that all at-risk populations can be adequately protected.

Ethiopia successfully implemented a Global Technical Strategy (GTS) to reduce malaria case incidence by 40% compared to 2015 by 2020 [2]. However, the subsequent GTS plan, which aimed at reducing malaria cases by 75% compared to 2015 by 2025, appears challenging. Therefore, a holistic, urgent intervention is required to combat this public health problem.

## 5. Limitations of the Study

Since we used a cross-sectional study, the results cannot elucidate the causal and effect relationships between risk factors and *Plasmodium* infection. In addition, our study was carried out in a single health facility with a limited sample size, focusing solely on symptomatic patients. Moreover, there is potential underrepresentation of sociodemographic variables such as family income and educational status due to challenges in accurately collecting and analyzing these data. These limitations impact the generalizability and comprehensiveness of the findings. Despite the significance of our study, it would fail to provide a comprehensive picture of malaria prevalence in the community.

## 6. Conclusion

This study demonstrated a high malaria burden at elevations above 2000 m among Teda Health Center outpatients, with *P. vivax* being the dominant species. Individuals above the age of 15 years, rural residents, individuals who did not use LLIN and had a family member with a history of malaria were at higher risk of getting malaria. The identified malaria infection determinants could be controlled using health education for protection against mosquito bites, encouraging early treatments, and environmental management. In addition, the distribution of LLIN and IRS should be reinforced. Moreover, awareness is warranted about vector control strategies targeting individuals engaged in outdoor activities and who reside near surface waters to sustain the achievements made previously. Despite the limitations of our study, the findings hold significance for broader application within communities sharing similar environmental characteristics. Our study suggests that areas with similar environmental conditions, like higher altitude and more cases of *P. vivax* infection, could benefit from our findings.

## Figures and Tables

**Table 1:** Sociodemographic characteristics of the study participants attending Teda Health Center, Northwest Ethiopia (*n* = 318).

Sociodemographic characteristics		Frequency	Proportion (%)
Age groups (in years)	<5	43	13.5
	5–15	98	30.8
	>15	177	55.7

Sex	Male	173	54.4
	Female	145	45.6

Family size	<5	114	35.8
	≥5	204	64.2

Residence	Urban	137	43.1
	Rural	181	56.9

**Table 2:** Clinical manifestations of the study participants attending Teda Health Center, Northwest Ethiopia (*n* = 318).

Clinical characteristics		Age groups in year, *n* (%)			
		<5	5–15	>15	Total
Headache	Yes	30 (69.8)	90 (91.8)	169 (95.5)	289 (90.9)
	No	13 (30.2)	8 (8.2)	8 (4.5)	29 (9.1)

Fever	Yes	42 (97.7)	75 (76.5)	79 (44.6)	196 (61.6)
	No	1 (2.3)	23 (23.5)	98 (55.4)	122 (38.4)

Sweating	Yes	35 (81.4)	45 (45.9)	59 (33.3)	139 (43.7)
	No	8 (18.6)	53 (54.1)	118 (66.7)	179 (56.3)

Chills	Yes	26 (60.5)	56 (57.1)	83 (46.9)	165 (51.9)
	No	17 (39.5)	42 (42.9)	94 (53.1)	153 (48.1)

Loss of appetite	Yes	43 (100.0)	97 (99.0)	148 (83.6)	288 (90.6)
	No		1 (1.0)	29 (16.4)	30 (9.4)

Nausea	Yes	43 (100.0)	89 (90.8)	130 (73.4)	262 (82.4)
	No		9 (9.2)	47 (26.6)	56 (17.6)

Vomiting	Yes	40 (93.0)	39 (39.8)	32 (18.1)	111 (34.9)
	No	3 (7.0)	59 (60.2)	145 (81.9)	207 (65.1)

Weakness	Yes	43 (100.0)	94 (95.9)	166 (93.8)	303 (95.3)
	No		4 (4.1)	11 (6.2)	15 (4.7)

Joint pain	Yes	17 (39.5)	32 (32.7)	89 (50.3)	138 (43.4)
	No	26 (60.5)	66 (67.3)	88 (49.7)	180 (56.6)

**Table 3:** Individuals, housing, and environmental factors contributing to the transmission of malaria at Teda Health Center, Northwest Ethiopia (*n* = 318).

Variables	Category	Frequency	Percent
Malaria history with in the previous 1 year	Yes	106	33.3
	No	212	66.7

Presence of family members with history of malaria	Yes	130	40.9
	No	188	59.1

Knowledge about malaria transmission	Yes	158	49.7
	No	160	50.3

Habit of sleeping or working outside at night	Yes	78	24.5
	No	240	75.5

LLIN utilization	Yes	97	30.5
	No	221	69.5

Presence of a hole on the house	Yes	117	36.8
	No	201	63.2

Presence of surface water within 500 m of the house	Yes	215	67.6
	No	103	32.4

**Table 4:** Bivariate and multivariate logistic regression analysis of factors associated with malaria infection among suspected outpatients at Teda Health Center, Northwest Ethiopia (*n* = 318).

Variables	Category	Total examined	Positive (%)	COR (95% CI)	AOR (95% CI)	*p*
Sex	Male	173	46.8%	2.088 (1.312-3.325)^∗^	1.233 (0.683-2.228)	0.487
	Female	145	29.7%	1		

Age group (in years)	<5	43	48.8%	1		
	5-15	98	45.9%	1.299 (0.784-2.152)	2.413 (0.905-6.436)	0.078
	>15	177	37.9%	2.976 (1.348-6.574)^∗^	2.704 (1.370-5.336)	0.004

Family size	<5	114	27.2%	1		
	≥5	204	50.0%	1.858 (1.141-3.024)^∗^	1.638 (0.874-3.073)	0.124

Residence	Urban	137	22.6%	1		
	Rural	181	56.4%	3.614 (2.203-5.929)^∗^	2.556 (1.281-5.098)	0.008

Knowledge about malaria transmission	Yes	184	34.2%	1		
	No	134	52.2%	1.498 (0.952-2.357)		

LLIN utilization	Yes	97	16.5%	1		
	No	221	52.9%	4.045 (2.274-7.196)^∗^	3.257 (1.633-6.499)	0.001

Malaria history within the previous 1 year	Yes	106	50.0%	2.673 (1.653-4.321)^∗^	1.579 (0.878-2.840)	0.127
	No	212	37.7%	1		

Presence of family members with history of malaria	Yes	130	58.5%	3.440 (2.145-5.519)^∗^	2.902 (1.671-5.041)	*p* ≤ 0.000
	No	188	30.3%	1		

Habit of sleeping or working outside at night	Yes	81	60.5%	4.006 (2.340-6.858)^∗^	1.988 (1.020-3.875)	0.044
	No	237	35.4%	1		

Presence of a hole on the house	Yes	117	58.1%	3.008 (1.872-4.835)^∗^	1.528 (0.818-2.854)	0.183
	No	201	32.3%	1		

Presence of surface water within 500 m of the house	Yes	215	49.8%	2.862 (1.685-4.861)^∗^	1.732 (0.901-3.331)	0.099
	No	103	25.2%	1		

AOR: adjusted odds ratio; CI: confidence interval; COR: crude odds ratio. ^∗^*p* < 0.05.

## Data Availability

The data for this study is obtainable from the corresponding author upon reasonable request.

## Abbreviations

AOR: Adjusted odds ratio
CI: Confidence interval
COR: Crude odds ratio
GTS: Global Technical Strategy
IRS: Indoor residual spraying
LLIN: Long-lasting insecticide net
WHO: World Health Organization.
